# Hydrogen Sulfide and Endoplasmic Reticulum Stress: A Potential Therapeutic Target for Central Nervous System Degeneration Diseases

**DOI:** 10.3389/fphar.2020.00702

**Published:** 2020-05-14

**Authors:** Huimin Zhong, Huan Yu, Junjue Chen, Jun Sun, Lei Guo, Ping Huang, Yisheng Zhong

**Affiliations:** ^1^Shanghai Key Laboratory for Prevention and Treatment of Bone and Joint Diseases, Shanghai Institute of Traumatology and Orthopaedics, Ruijin Hospital, Shanghai Jiao Tong University School of Medicine, Shanghai, China; ^2^Department of Ophthalmology, Ruijin Hospital Affiliated Medical School, Shanghai Jiaotong University, Shanghai, China

**Keywords:** hydrogen sulphide, central nervous system, degeneration disease, endoplasmic reticulum stress, neuroprotection

## Abstract

There are three members of the endogenous gas transmitter family. The first two are nitric oxide and carbon monoxide, and the third newly added member is hydrogen sulfide (H_2_S). They all have similar functions: relaxing blood vessels, smoothing muscles, and getting involved in the regulation of neuronal excitation, learning, and memory. The cystathionine *β*-synthase (CBS), 3-mercaptopyruvate sulfur transferase acts together with cysteine aminotransferase (3-MST/CAT), cystathionine *γ*-lyase (CSE), and 3-mercaptopyruvate sulfur transferase with D-amino acid oxidase (3-MST/DAO) pathways are involved in the enzymatic production of H_2_S. More and more researches focus on the role of H_2_S in the central nervous system (CNS), and H_2_S plays a significant function in neuroprotection processes, regulating the function of the nervous system as a signaling molecule in the CNS. Endoplasmic reticulum stress (ERS) and protein misfolding in its mechanism are related to neurodegenerative diseases. H_2_S exhibits a wide variety of cytoprotective and physiological functions in the CNS degenerative diseases by regulating ERS. This review summarized on the neuroprotective effect of H_2_S for ERS played in several CNS diseases including Alzheimer’s disease, Parkinson’s disease, and depression disorder, and discussed the corresponding possible signaling pathways or mechanisms as well.

## Introduction

Hydrogen sulfide (H_2_S) plays an important role in terms of cell signal transduction and modulation in the central nerve system (CNS), the cardiovascular system, and many organs like hepatic function (Olas, 2015; Wu et al., 2019). H_2_S shows its function on inflammatory cells, endoplasmic reticulum, and mitochondria so that H_2_S may promote resolution of inflammation, energy metabolism, mitochondrial function and misfold protein (Sivarajah et al., 2009; Polhemus and Lefer, 2014; Wallace et al., 2018). Moreover, neuronal signaling mediated by H_2_S contributes to neuromodulation properties and neuroprotection because H_2_S concentration distribution in the brain is up to threefold higher than in serum (Zhao et al., 2001; Hogg, 2009; Paul and Snyder, 2018). Many different donors of H_2_S to cells are widely used in scientific research. Endogenous H_2_S is difficult to accurately survey due to low concentration in the body. Therefore, the biological effects of H_2_S can be simulated by injecting exogenous gas into cells or donors *in vitro* (Powell et al., 2018). Sodium hydrosulfide (NaHS) and sodium sulfide have been exerted to give a burst of H_2_S but short duration (Cheung et al., 2007). GYY4137, AP97, and AP105 can trigger the corresponding release of H_2_S *in vivo*. Another advantage is that they can target organelles (Li and Moore, 2007; Li et al., 2011; Hancock and Whiteman, 2014). The article reviews the effects of H_2_S on the endoplasmic reticulum stress (ERS) pathway in the pathogenesis of neurodegenerative and psychiatric diseases.

## H_2_S in CNS

As is known to all, H_2_S is a colorless, water-soluble, highly toxic acidic gas with a depressing smell of rotten eggs (Xiao et al., 2018). H_2_S is slightly soluble at the physiological condition of 37°C , pH 7.4 and pK1 = 6.76; four-fifths of H_2_S is dissociated form (HS^-^ and S^2-^), and less than one-fifths is undissociated form (H_2_S). At PH 6.0, H_2_S mainly exists as gas (Ishigami et al., 2009). However, H_2_S, an endogenously produced gas, also has been qualified as the new third gas transmitter, signaling molecule, antioxidants, antiapoptotic agents and nerve cell protectant (Kumar and Sandhir, 2018). H_2_S exerts its function in maintaining a balance between oxidation and antioxidant to protect neurons from oxidative stress (Shefa et al., 2018). The role of H2S in ischemic brain depends on the concentration; low concentrations have a protective effect, and high concentrations do the opposite (Chan and Wong, 2017).

H_2_S is produced from cysteine by enzymes. There are four enzymes responsible for endogenous H_2_S generation: Cystathionine *β*-synthase (CBS), 3-mercaptopyruvate sulfur transferase acts together with cysteine aminotransferase (3-MST/CAT), cystathionine *γ*-lyase (CSE), and 3-mercaptopyruvate sulfur transferase with D-amino acid oxidase (3-MST/DAO) pathways (Chen et al., 2015). The first three utilize L-cysteine or homocysteine as substrates. While the 3-MST/DAO pathway is a novel source of endogenous H_2_S; the substrate is the less toxic D-cysteine (Shibuya et al., 2013). D-cysteine would be more advantageous than L-cysteine as a neuroprotectant against cerebellar ataxias. Furthermore, their distribution is highly tissue specific. The three enzymes CBS, 3-MST/CAT, and 3-MST/DAO are mainly localized in the brain, while the fourth enzyme CSE produces H_2_S in other organs. Additionally, the 3-MST pathway functions as the main producer to release H_2_S in the brain. Additionally, the 3-MST pathway functions as the main producer of H_2_S and polysulfides (H_2_S_n_) in the brain. 3-Mercaptopyruvate (3-MP), the substrate of 3-MST, can produce protein-bound polysulfides so that H_2_S_n_ generated by 3-MST exists in the brain (Hylin and Wood, 1959; Kimura, 2019). In the meantime, H_2_S in CNS parts such as the hippocampus, brain stem, cerebellum, and brain is commonly generated by CBS as well (Chen et al., 2015). CSE is largely associated with peripheral or nonnervous tissues. Thus, H_2_S produced by CBS and 3-MST/CAT is mainly discussed.

### Production of H_2_S and H_2_S_n_

There are two possibilities for the mechanism of H_2_S releasing. One is a nonenzymatic pathway that is released immediately once H_2_S is produced. The other possibility is the enzymatic pathway that releases H_2_S storage produced by enzyme under specific conditions (Ishigami et al., 2009; Zaorska et al., 2020). As mentioned above, H_2_S production is closely related to enzymes. CBS and CSE comprise the transsulfuration pathway and also have the ability to catalyze the desulfhydration of cysteine. Relatively, 3-MST that is mostly located in the mitochondria gets involved in the cysteine catabolic pathway (Banerjee et al., 2015). Distribution of enzymes is highly tissue specific although all of them can be detected in many organs. Representative enzyme in the brain is CBS followed by 3-MST, whereas CSE is the most active in the cardiovascular system (Bao et al., 1998; Yang et al., 2008).

**Figure 1:**
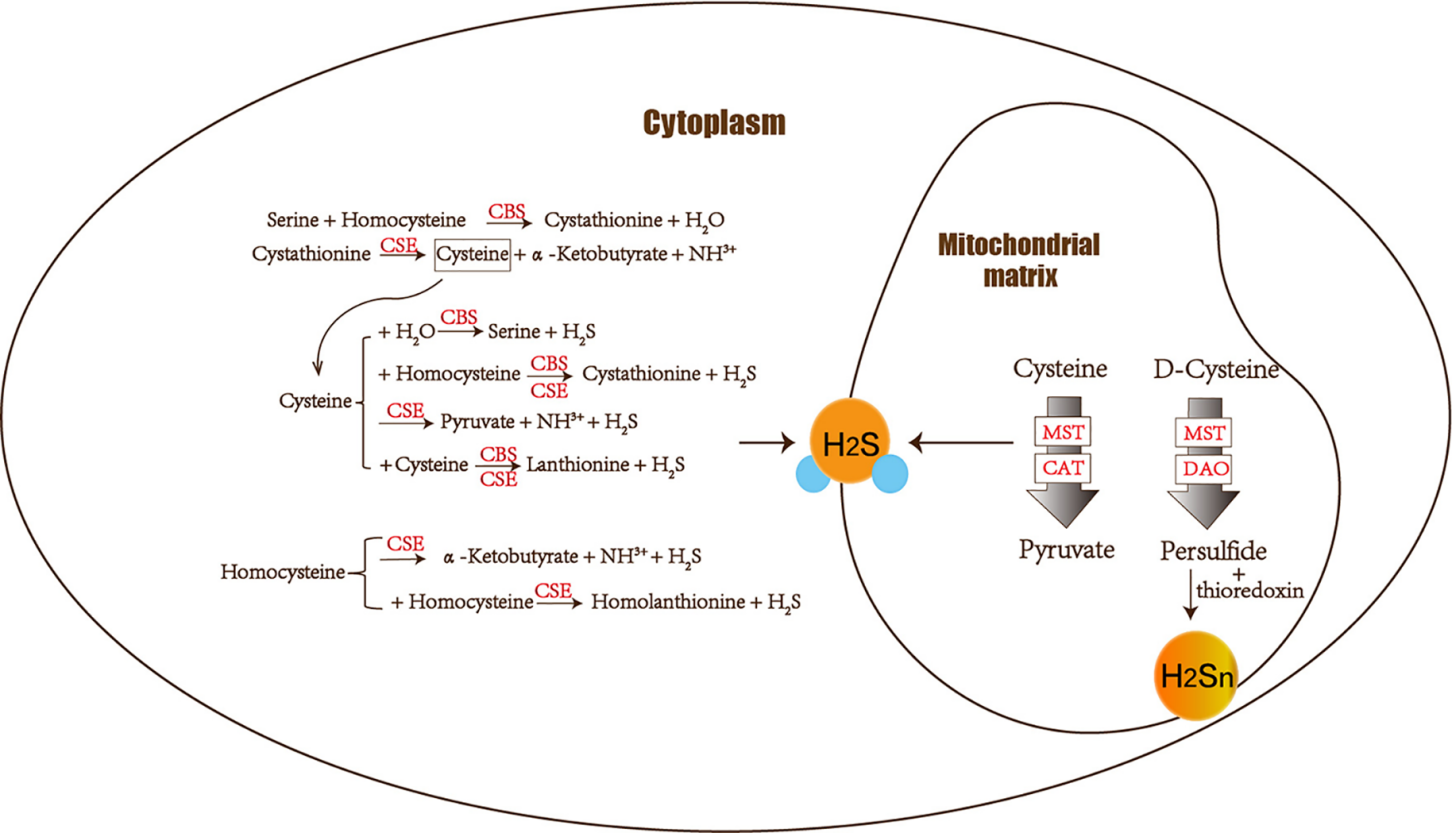
The figure shows the enzymes involved in H_2_S biogenesis. Although enzymes can be present in the cytoplasm and mitochondria, H_2_S produced by MST dominates the mitochondria. CBS, CSE, MST/CAT, and MST/DAO represent cystathionine *β*-synthase, cystathionine *γ*-lyase ; mercaptopyruvate sulfur transferase acts together with cysteine aminotransferase and mercaptopyruvate sulfur transferase with D-amino acid oxidase. The 3-MST/DAO pathway also can produce persulfide, which interacts with thioredoxin to release H_2_S_n_.

The amino acids cysteine and homocysteine have been qualified as the sulfur source. Under the catalysis of CBS, homocysteine and serine undergo a replacement reaction, and the products are water and cystathionine. Next is the elimination reaction dominated by CSE, which produces cysteine, *α*-ketobutyrate and NH^3+^. This series of reactions completes the conversion of the harmful substance homocysteine to cysteine. Then CSE and CBS achieve condensation of homocysteine and cysteine in common. Furthermore, homocysteine itself can also generate H_2_S under the catalysis of CSE enzyme. Cysteine plays a crucial role in diverse H_2_S-producing reactions, which can complete replacement and cracking reaction of different objects under the catalysis of CBS and CSE. CBS catalyzes the *β*-replacement of cysteine + homocysteine or cysteine + H_2_O to liberate H_2_S. Similarly, in addition to participating in the cracking reaction of cysteine + homocysteine and the *γ*-replacement reaction of two moles of homocysteine, CSE also takes part in cysteine’s condensation reaction with itself to produce H2S (Banerjee et al., 2015) (**Figure 1**).

CAT also catalyzes the transamination between cysteine and *α*-ketoglutarate, but the products are mercaptopyruvate and glutamate. Subsequently MST forms persulfide and pyruvate to liberate H_2_S under reducing conditions (Yadav et al., 2013). MST is an enzyme that can transfer sulfur from mercaptopyruvate to make MST-SH into MST-SSH. MST-SSH is a persulfide intermediate that releases H_2_S in the presence of a reductant (Banerjee et al., 2015). H_2_S is produced from 3-MP by 3-MST. When synthesizing 3-MP in the mitochondria from cytoplasmic D-cysteine, it needs participation of the enzyme DAO (Ubuka et al., 1978; Shibuya et al., 2013) (**Figure 1**).

Hideo Kimura identified H_2_S_2_ and H_2_S_3_ produced by 3MST in the brain. The intermediate products persulfide or polysulfide generated after 3-MST catalysis interact with thioredoxin to release H_2_Sn (mainly H_2_S_2_) (Kimura, 2019). Most H_2_Sn-related enzymes are distinct from H_2_S. Copper/zinc superoxide dismutase can be utilized to produce H_2_S_2_, H_2_S_3_, and H_2_S_5_ (Searcy et al., 1995; Olson et al., 2018). Peroxidases such as lactoperoxidase and myeloperoxidase can oxidize H_2_S to polysulfides (Nakamura et al., 1984; Garai et al., 2017). Two gas signal molecules NO and H_2_S will also interact and produce H_2_S_n_ (Eberhardt et al., 2014; Moustafa and Habara, 2016). H_2_S_n_ (n ≥ 2) induces Ca^2+^ influx in astrocytes more effectively than H_2_S (Kimura et al., 2013). Neurons can be protected by H_2_S and H_2_S_n_ (such as H_2_S_4_) to reduce the damage of oxidative stress (Kimura et al., 2019). As a result, both H_2_S and H_2_S_n_ play a role in neurodegenerative diseases.

### H_2_S Is Produced by CBS

CBS is an enzyme that depended on pyridoxal-5′-phosphate, and CBS has been found in the hippocampus, brainstem, cerebellum, cerebrum. CBS can produce efficient H_2_S through the *β* displacement condensation reaction of cysteine and homocysteine *β* substitution (Chen et al., 2004). CBS is of great importance to regulate homocysteine levels *in vivo* because mice lacking CBS behave hyperhomocysteinemia and hypermethioninemia (Ishii et al., 2010). Endogenous H_2_S in the brain is generated mainly by CBS so that the change of H_2_S level depends on altering CBS expression. CBS mRNA expression or CBS transcription can be increased under the regulation of epidermal growth factor, transforming growth factor-α, and cyclic adenosine monophosphate (CAMP). Activation of astrocytes and microglia inflammatory pathways in neuronal cells will reduce CBS expression, leading to downgrade H_2_S levels in the brain (Schicho et al., 2006; Kimura et al., 2010).

Under local oxidizing conditions, the ferrous form of CBS is less active than the ferric form (Taoka et al., 1998). Due to the very low chemical reaction potential of Fe^3+^/Fe^2+^ in CBS (−350mV), the availability of ferrous states in CBS is unclear (Kabil et al., 2011). The CO system will interfere with the H_2_S system, a novel member of the gas-transmitter family, because CO in the ferrous state will combine with CBS to inhibit the activity of CBS (Shintani et al., 2009). S-adenosylmethionine is the precursor of homocysteine, which can activate CBS by binding to the carboxy-terminal domain of CBS, thus H_2_S also increases (Sen et al., 2012). CBS expression is abnormal in several diseases. CBS level is found to be threefold higher in the brains of Down’s syndrome patients than in the normal people, but CBS allele expression is lower in children with high intelligence quotient (Kimura, 2010). Experiments have shown that L-glutamic acid and calcium (Ca^2+^)/calmodulin in hippocampal slices of rats can promote and maintain the production of H_2_S by CBS, respectively. N-Methyl-D-aspartate (NMDA) is involved in this process as well because CBS functions as an antioxidant inhibitory *via* triggering NMDA receptors. One of the reasons for the high H_2_S concentration in the cerebrospinal fluid of patients with Down’s syndrome may be that the addition of NMDA receptors has altered the long-term enhancement of the hippocampus. Encoded and overexpressed CBS on chromosome 21 in these patients causes this increased H_2_S concentration. What’s more, H_2_S level of the patients with Alzheimer disease reduces 55%. Studies show that continuous leaks of H_2_S can impair fetal neuronal development and monoamine neurochemistry in experimental rats (Cheung et al., 2007).

Thus, H_2_S is very relevant to neurodegenerative diseases. In a word, overexpression and deficiency of CBS both lead to serious diseases such as cognitive dysfunction. If we can understand how to balance the expression of CBS in the brain, the potential therapeutic pathways for many central nervous system diseases can be expanded.

### H_2_S Is Produced by 3-MST/CAT

It has been reported that 3-MST/CAT induces H_2_S in the brain and mostly get involved in the neuronal generation of H_2_S (Kimura et al., 2010; Panthi et al., 2016). CBS is mainly localized in the cytosolic of cells, while 3-MST is found in the mitochondrial matrix of neurons in the brain and retina (Nagahara et al., 1998; Shibuya et al., 2009a; Shibuya et al., 2009b; Mikami et al., 2011a; Mikami et al., 2011b).

It has recently been shown that substrates containing L-cysteine and D-cysteine in the brain can produce H_2_S through the 3-MST/CAT and 3-MST/DAO pathways (Panthi et al., 2016). Thioredoxin and dihydrolipoic acid (DHLA) integrate with 3-MST to release H_2_S. The concentration of DHLA in the brain is approximately 40 μM, which enhances the H_2_S production effectively (Mikami et al., 2011a; Mikami et al., 2011b). Actually, 3-MP is the substrate of the mitochondrial enzyme 3-MST, which itself also acts as an endogenous H_2_S donor (Mitidieri et al., 2018). The mitochondria are the intracellular storage form of H_2_S. 3-MP stimulated mitochondrial H_2_S production and enhanced mitochondrial electron transport at low concentrations (Modis et al., 2013). L-cysteine and α-ketoglutarate provide 3-MP by the CAT pathway. When synthesizing 3-MP in the mitochondria from cytoplasmic D-cysteine, it needs participation of enzyme DAO (Ubuka et al., 1978; Shibuya et al., 2013). DAO is rich in cerebellar tissues which can convert D-cysteine to 3MP, thereby effectively producing H_2_S. H_2_S generated by D-cysteine exerts its function in promoting dendritic development of cerebella Purkinje cells (Seki et al., 2018). H_2_S produces from 3-MP by 3-MST, so unstable molecule 3-MP is also the intermediate of CAT catalysis that affects the formation of 3-MST (Shibuya et al., 2009b; Mikami et al., 2011a). Under the presence of dithiothreitol (DTT), persulfide can be produced to release H_2_S through the way of providing sulfur by 3-MP at the active site of 3-MST (Shibuya et al., 2009b; Kabil and Banerjee, 2010).

On the other hand, Ca^2+^ concentration is closely related to the production of H_2_S. The production of H_2_S is the highest when the Ca^2+^ concentration is zero and minimum under the condition of 2.9 μM Ca^2+^ so that the activity of CAT is inhibited by the Ca^2+^ concentration as well (Mikami et al., 2011b). Mikami et al. also found that 3-MST produced H_2_S from thiosulfate. In the presence of high concentrations of DHLA, H_2_S can be produced from both 3MP and thiosulfate. They also concluded that DHLA was detected to release H_2_S effectively from the brain post-nuclear supernatant containing bound sulfane sulfur (Mikami et al., 2011a).

There are three primary biological forms of H_2_S including free, acid-labile, and bound sulfur. The acid-labile sulfur is another form of the sulfur pool to release H_2_S, which is primarily located at the iron–sulfur cluster of enzymes in the mitochondria. Experiments have shown that acid-labile sulfur can be discovered in the brains of rats and humans by detecting the shape of different sulfides. H_2_S can be released from acid-labile sulfur at acidic conditions while from bound sulfur in alkaline microenvironment. The highest PH for H_2_S to release from acid-labile sulfur is 5.4. In fact, the acid-labile sulfur releasing H_2_S pathway may be difficult because the mitochondria are usually not in the acidic environment (Ishigami et al., 2009). Bound sulfur is localized to the cytoplasm and acts as an intracellular storage of H_2_S. Because 3-MST cell expression mutation lacks H_2_S-producing activity, the concentration of bound sulfur is low (Shibuya et al., 2009b). 1,500 nmol/g protein concentrations of bound sulfur can release enough H_2_S to stimulate the target molecules in the brain. When neurons are excited, the increase of extracellular potassium ion concentration causes the intracellular pH of astrocytes to increase to release H_2_S from the bound sulfur (Ishigami et al., 2009). Moreover, another substrate for CAT, aspartate can combine competitively with CAT to suppress the production of H_2_S. 3-MST generates bound sulfur more efficiently than CBS. 3-MST is more active than CBS to transfer bound sulfur from H_2_S (Kimura, 2010).

However, specific proteins that can be used to identify whether H_2_S releases physiological or pathological signals from the storage form are still unknown.

### H_2_S as a Signaling Molecule in the CNS

H_2_S plays a significant role in regulating the function of the CNS as a signaling molecule. It has been found that H_2_S is involved in neuroprotection processes and neurotransmission in various models (Nagai et al., 2004; Hu et al., 2008; Qu et al., 2008). The potential mechanism of neuroprotection of H_2_S contains anti-inflammation and upregulation of antioxidative enzymes. H_2_S may protect neurons from apoptosis and degeneration as well (Popov, 2013). H_2_S also plays a neuroprotective function by regulating the intracellular pH of microglia and restricting the injury of activated microglia in the damaged site (Lu et al., 2010). H_2_S results in immoderate NMDA receptor stimulation through the transmitter cAMP. One of the endogenous ligands of the NMDA receptors is glutamic acid. The function of protein kinase A (PKA) is to make various intracellular proteins phosphorylation and to get involved in maintaining brain activity. After the addition of the glutamate receptor subunit, phosphorylation happens in the NMDA receptor 1 ion channel by activation of cAMP-dependent PKA (Zhao et al., 2016). Thus, H_2_S may influence the behaviors of NMDA receptors and second messenger systems through changing intracellular cAMP levels and increases intracellular Ca^2+^ by activating voltage-gated sodium channels in neuronal cells (Zhang and Bian, 2014). Generation of cAMP stimulates PKA, thereby phosphorylating various intracellular proteins, and an influx of Ca^2+^ ions is observed in this process (Tan et al., 2010). Studies have shown that CAMP of the primary cultures of the cerebral cortex, cerebellar neurons, and glial cells increases because of the enhanced concentration of NaHS sustained release H_2_S (Kimura, 2000). Therefore, H_2_S may modulate intracellular cAMP levels and thereby activate PKA to regulate the activity of NMDA receptors. At the same time, H_2_S may increase intracellular Ca^2+^ concentration by activating sodium channels on neuronal cell membranes (Kimura, 2011).

Research shows that H_2_S protects neurons from oxidative stress. Hypochlorous acid (HOCl) or peroxynitrite (ONOO^−^) interacts with tyrosine to form 3-chlorotyrosine, which is toxic to neurons. H_2_S has the function of restraining the activity of reactive oxygen species (ROS), reactive nitrogen species (RNS) or chlorine (such as HOCl), so it can significantly inhibit HOCl toxicity by eliminating HOCl before neurodegenerative changes occur (Olas, 2017). H_2_S protects neuroblastoma cells from oxidative/nitrative stress induced by ONOO^−^ or HOCl (Whiteman et al., 2004). In addition, H_2_S may protect retinal neurons from light-stimulated degeneration (Mikami et al., 2011b; Kimura, 2014).

ONOO^−^ can nitrate phenolic groups of tryptophan and tyrosine in proteins, and 3-nitrotyrosine is formed consequently. Due to the toxicity of ONOO^−^ itself and its products to neuronal cells, it plays a role in neurodegenerative diseases such as Alzheimer’s disease, Huntington’s disease, and Parkinson’s disease or amyotrophic lateral sclerosis. Reduced H_2_S levels in these diseases lead to increasing ONOO^−^ activity and neuronal degeneration. H_2_S exerts a protective function by inhibiting the interaction between ONOO^−^ and tyrosine (Cheung et al., 2007). Rho-associated protein kinase 2, a key factor that promotes neurodegeneration in Parkinson’s disease, can be reduced by H_2_S through microRNA-mediated protection of nerve cells (Liu et al., 2016). Persulfidation of H_2_S signals actually modifies cysteine residues on target protein and converts SH group to SSH group. Dysregulation of the transsulfuration pathway which generates H_2_S occurs in several neurodegenerative diseases (Paul and Snyder, 2018). Sulfhydration might be impaired in protein misfolding diseases such as Parkinson’s disease. H_2_S inhalation has been studied for its neuroprotective action in a tested mouse model of Parkinson’s disease (Kida et al., 2011). Administration of H_2_S donor in the APP/PS1 mouse model can attenuate cognitive dysfunction caused by homocysteine. H_2_S can inhibit the oxidative stress pathways that affect AD. The generation of oxidative stress markers homocysteine-induced malondialdehyde (MDA) and 4-hydroxynonenal (4-HNE) can be reduced by H_2_S. Nrf2 plays a key role in maintaining redox balance so that NaHS (one of H2S donors) can increase the expression of Nrf2. As a result, H_2_S mediates neuroprotection *via* multiple pathways (Paul and Snyder, 2018).

Everything has two sides. Steady intracellular concentrations of H_2_S depend on enzymatic generation and clearance rates (Kabil and Banerjee, 2014). H_2_S is also a two-edged sword that excessive H_2_S can have an adverse result. The inflammatory factor interleukin-1*β* (IL-1*β*) causes memory loss through stimulating the CBS enzyme. H_2_S-mediated IL-1*β* results in degradation of postsynaptic density 95 which is an important scaffold protein to promote synapse maturation. The loss of postsynaptic density 95 implicates in brain diseases such as consequent neuronal spine retraction (Mir et al., 2014). CBS level is found to be threefold higher in the brains of Down’s syndrome patients than in the normal people but CBS allele expression is lower in children with high intelligence quotient. Overexpression of CBS is present on the trisomy of chromosome 21 (Kimura, 2010). CBS is localized to astrocytes adjacent to the senile plaques in the brains of Down’s syndrome patients (Paul and Snyder, 2015).

H_2_S production and metabolism should be balanced and regulated in the nervous system. It should be focused on uncovering the exact role and function of H_2_S in the CNS with the aims of dissecting the involved signaling pathways.

## ERS of Neurons in CNS

In eukaryotic cells, the endoplasmic reticulum (ER) is an organelle that synthesizes, modifies, and folds proteins into the correct structure. Only correctly folded proteins can be transported to the Golgi apparatus for further processing. When the protein load capacity of ER is insufficient to deal with unfolded or misfolded proteins accumulated in the ER, the ER balance will be broken and the Ca^2+^ balance will be disturbed, resulting in endoplasmic reticulum stress (ERS). To alleviate this stress state, the cell firstly initiates an unfolded protein response (UPR), a self-protection mechanism, to eliminate unfolded protein stacks and facilitate cell survival. UPR is mediated by glucose-regulated protein 78 (GPR78)/immunoglobulin heavy chain binding protein (Bip), and three ERS-sensing proteins located on the ER membrane. The three ERS-sensing proteins are double-stranded RNA-dependent protein kinase (PKR)-like ER kinase (PERK) and type-1 ER transmembrane protein kinase (IRE-1) and activating transcription factor 6, ATF6 (Saito, 2014). Although ERS can activate UPR, a self-protection mechanism, severe ERS can still induce apoptosis and death (Cabral Miranda et al., 2014). Short-term activation of UPR is protective, but sustained UPR stimulation caused by prolonged ERS duration can promote neurotoxicity. Protein misfolding and trafficking in the ER lumen initiate UPR and cause the toxic accumulation in energy-starved neurons. Chorionic activation of ERS plays an important role in various neurological diseases, mainly neurodegenerative diseases, followed by spinal cord injury, sclerosis and diabetic nerves (Zhang et al., 2015).

Under normal circumstances, the three inactive proteins are bound to GPR78/Bip, respectively. When the ER homeostasis is broken and a large amount of unfolded proteins accumulate in the ER, GPR78/Bip dissociates from the three sensory proteins in order to bind the accumulated unfolded proteins, releasing and activating the three sensory proteins, thereby activating UPR (Almanza et al., 2019). The role of GRP78 is mainly to regulate the initiation of UPR through direct interaction with each sensory protein (Bertolotti et al., 2000; Shen et al., 2002). After dissociation of PERK and GPR78/Bip, the downstream eukaryotic initiation factor 2*α* (eIF2*α*) is subsequently phosphorylated. Phosphorylated eIF2*α* restricts unfolded proteins from entering the ER, which is beneficial to cell survival; in addition, phosphorylated eIF2*α* can regulate the expression of activated transcription factor 4 (ATF4) and participate in the recovery of protein synthesis. After IRE-1 is activated, X-box binding protein-1 (XPB-1) is further activated. Activated XBP-1 accelerates the degradation of ER-related proteins, repairs the steady state of endoplasmic reticulum, and plays a role in cell protection. Dissociation of ATF6 from GPR78/Bip can activate the molecular chaperone Bip, the ER protein target gene and upregulate XBP-1, helping the protein to fold, modify, and transport correctly, thereby maintaining cell homeostasis. However, under the stimulation of continuous or excessively strong ERS, UPR cannot continue to maintain homeostasis in the ER, leading to CCAAT-enhancer-binding protein homologous protein (CHOP) activation. Activated CHOP disrupts the balance between downstream apoptotic genes and finally indirectly induces apoptosis (Hetz and Papa, 2018).

Features of pathophysiological stress induced by ERS like protein aggregates, inflammatory signals, metabolic alterations trigger UPR. The changes in the UPR pathway due to ERS may work in the pathogenesis of diabetic neuropathy (Sims-Robinson et al., 2012). Data indicated that icariin reduced neuronal apoptosis and suppressed ERS signaling including decreased the level of GRP78, phosphorylated ER-regulated kinase and phosphorylated eIF2α, as well ATF4, CHOP, DNA damage inducible protein 34 and tribbles homologous protein 3 to protect against Alzheimer’s disease animal model (Li et al., 2019). Carboxyl-terminus of the Hsp70 interacting protein (CHIP) prevents severe ERS-induced hippocampal neuron death. Experiments have shown that overexpression of CHIP prevents upregulation of both ERS-induced CHOP and p53 pro-apoptotic pathways and does not prevent growth of UPR-induced GRP78/Bip. Therefore, it is shown that overexpression of CHIP can weaken the ERS-induced apoptotic response while maintaining ERS adaptive changes in CNS (Cabral Miranda et al., 2014).

ROS and RNS disrupt correct protein folding structure in the ER lumen. Often, cells respond to oxidative stress by initiating ERS response. To human immunodeficiency virus (HIV)-associated neurocognitive impairment, Bip expression in HIV-positive cortex significantly increases and the cell specificity of the Bip level significantly increases in neurons and astrocytes. For the same group of patients, the expression of ATF-6*β* also upregulates (Lindl et al., 2007). Additionally, ERS is closely relevant to cell death and inflammatory signals. ERS induces astrocytes and neurons to secrete molecules with lipid characteristics. This molecule is a cascade reaction, which in turn regulates other astrocytes and neurons’ inflammation and ERS responses. Methylmercury enhances ERS levels to exert its toxicity through the inactivated Akt pathway mediated by ROS, thereby inducing neuronal apoptosis and eventually leading to death (Chung et al., 2019). Trace metals such as zinc (Zn), copper (Cu), and nickel (Ni) are essential in various physiological functions and have powerful biological functions. They are involved in the metabolic processes of enzymes, hormones, vitamins, and nucleic acids, but their excessive amounts cause disorders in various tissues of the CNS. Cu^2+^ markedly enhances Zn^2+^-induced neuronal cell death by activating ERS response. Excess Ni^2+^ can trigger the ERS response, which significantly enhances Zn^2+^-induced neuronal cell death, especially the expression of CHOP (Tanaka et al., 2019). H_2_S donor may be beneficial not only for the brain but also for spinal cord injury recovery *via* the ERS pathway. Cell autophagy induced by spinal cord injury can be remarkably blocked by the ERS inhibitor. From this, whether H_2_S can help ERS pathway against autophagy in CNS or not would be an interesting research (Wang et al., 2018). By targeting ERS molecular signaling responses, there will be more new perspectives on the protection and function maintenance of CNS neurons.

## The Effect of H_2_S on ERS of Neurons in CNS

### Neuroprotective Effects of H_2_S in Alzheimer’s Disease

**Figure 2:**
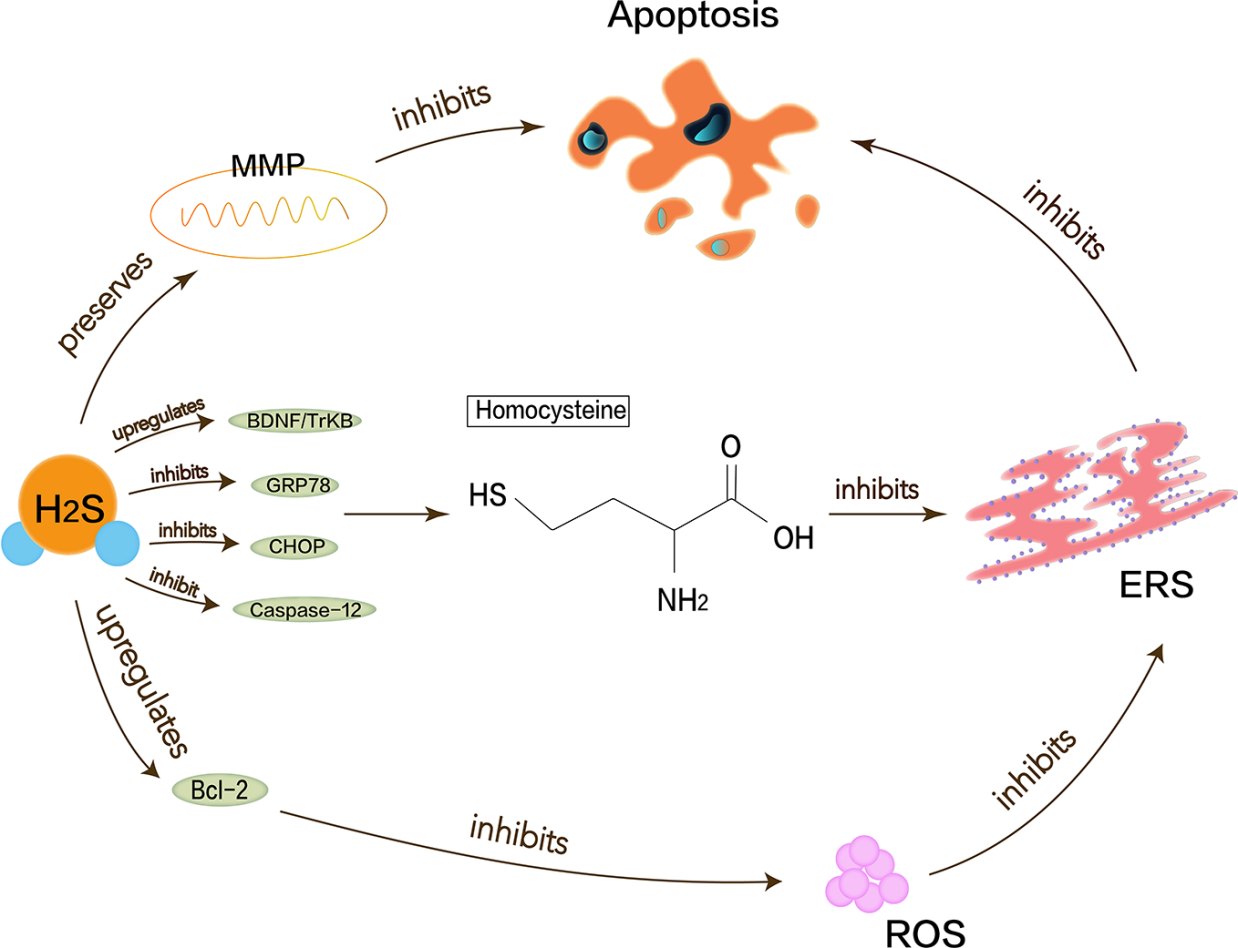
Neuroprotective effects of H_2_S in Alzheimer’s disease. Homocysteine can increase neuronal cell apoptosis and inhibit the production of endogenous H_2_S and cause the upregulation of ERS-related GRP78, CHOP, and cleaved caspase-12. Proper H_2_S concentration protects neurons by inhibiting ROS generation and preserving MMP pathway to reduce Alzheimer’s disease symptoms. H_2_S can increase expression of anti-apoptotic Bcl-2 or reduce cellular ROS toxicity and also restrain homocysteine-induced ERS and hippocampal neuronal apoptosis by upregulating BDNF/TrkB pathway in Alzheimer’s disease rat models.

Alzheimer’s disease (AD) is one of the most common destructive and progressive neurodegenerative diseases in the elderly. It mainly affects CNS, cognition, memory, and optic nerve abnormalities. The pathological manifestation is the presence of *β* amyloid deposits in the brain. Endogenous H_2_S may be closely related to the pathogenesis of AD because of the disordered H_2_S levels in the serum of AD patients. Proper H_2_S concentration protects neurons by inhibiting ROS generation and preserving the mitochondrial membrane potential (MMP) pathway (Tang et al., 2008). Hyperhomocysteinemia is a closely independent risk factor for AD because homocysteine can increase neuronal cell apoptosis and inhibit the production of endogenous H_2_S. Moreover, homocysteine causes the upregulation of ERS-related GRP78, CHOP, and cleaved caspase-12 (Wei et al., 2014). Studies have shown that NaHS interference in animal models of hyperhomocysteinemia can attenuate DNA damage and death of apoptotic cells to prevent neurodegeneration (Kumar et al., 2018). H_2_S also enhances the expression of anti-apoptotic Bcl-2 or reduce cellular ROS toxicity to protect homocysteine-induced cytotoxicity and apoptosis. Some evidence shows that H_2_S plays a key role in ERS pathology of AD. Experiments have demonstrated that H_2_S can attenuate the learning and memory decline in AD and inhibit the hippocampal ERS in homocysteine-exposed rats by reducing the expression of GRP78, CHOP, and cleaved caspase-12 (Zou et al., 2017). H_2_S can also restrain homocysteine-induced ERS and hippocampal neuronal apoptosis by upregulating the brain-derived neurotrophic factor/tropomyosin-related kinase B (BDNF/TrkB) pathway in AD rat models (Tang et al., 2010). NaHS releases endogenous H_2_S *in vivo* and increases the expression of BDNF in a dose-dependent manner, thereby significantly reducing homocysteine-induced apoptosis in ERS and hippocampal neurons. The protective effects of NaHS against homocysteine-induced ERS disappear when using k252a, a specific antagonist of TrkB (Wei et al., 2014). In brief, it is valued that H_2_S has a neuroprotective effect in AD, and ERS with its related pathways as shown in **Figure 2** should be referenced.

### Neuroprotective Effects of H_2_S in Parkinson’s Disease

Parkinson’s disease (PD) is the second most devastating progressive neurodegenerative disease after AD, with characteristic motor symptoms such as resting tremor and muscle stiffness. Broad and complex changes in neurons lead to Parkinsonian symptoms (Obeso et al., 2008).

Oxidative stress, mitochondrial dysfunction, neurotoxicity, neuroinflammation, and apoptosis have been considered as possible mechanisms that cause PD (Hirsch and Hunot, 2009). H_2_S is also closely relevant to PD. Neurotoxins such as 6-hydroxy-dopamine (6-OHDA) and 1-methyl-4-phenylpyridinium (MPP^+^) are used to simulate PD models *in vitro* and *in vivo*. MPP^+^ is the active metabolite of 1-methyl-4-phenyl-1,2,3,6-tetrahydropyridine (MPTP), which functions to stimulate the generation of superoxide radicals *in vitro* and induce cell apoptosis (Xiao et al., 2016). In a 6-OHDA-induced PD rat model, the endogenous H_2_S level of the primary lesion site of PD, substantia nigra (SN), is significantly reduced (Xue and Bian, 2015). Symptoms of PD may reflect compromised ubiquitylation. Neuroprotective ubiquitin E3 ligase of parkin in the brain of PD patients inactivates. and sulfhydration of parkin diminishes, while the persulfidation of parkin protein promotes the activity of ubiquitin E3 ligase, thereby mediating cell protection. H_2_S may upregulate the expression of deubiquitinating enzymes USP8 to antagonize the degradation of Parkin protein (Sun et al., 2020). This implies that H_2_S donors may be potentially therapeutic (Vandiver et al., 2013).

It turns out that the accumulation of misfolded or damaged proteins is related to the development mechanism of PD. The growth of mitochondrial dysfunction and the imbalance of oxidation and antioxidant will lead to the ER overload response that is the generation of ERS (Sarkar et al., 2016). Mitochondrial dysfunction and the imbalance of oxidation and antioxidant activate the pro-apoptotic pathway and inhibit the anti-apoptotic pathway. It is also proposed that mitochondrial dysfunction changes the energy-dependent cell membrane potential to generate free radicals and has toxic damage to cells (Sarkar et al., 2016). Excessive ROS can cause oxidative stress.

**Figure 3:**
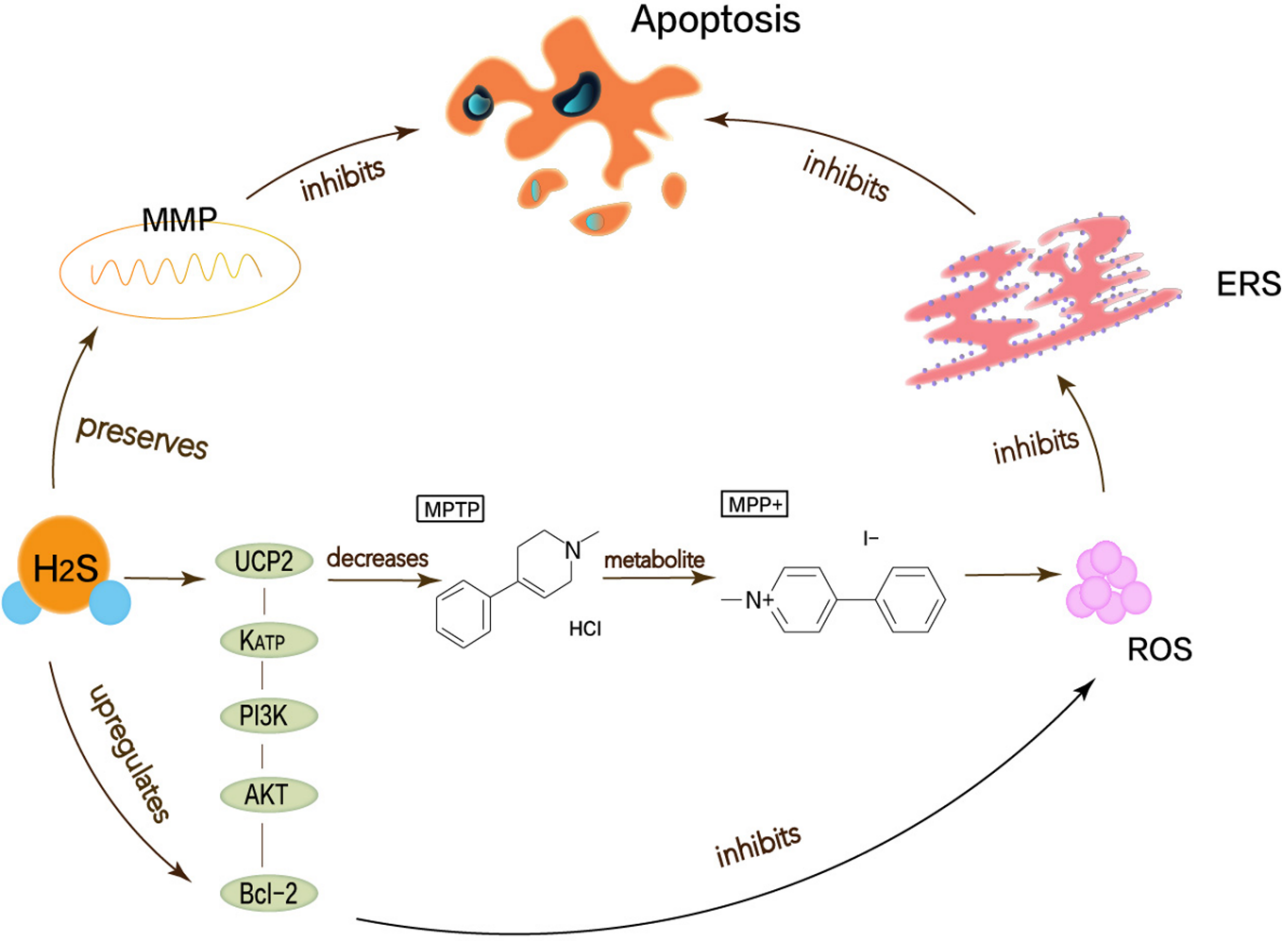
Neuroprotective effects of H_2_S in Parkinson’s disease. Oxidative stress, mitochondrial dysfunction, apoptosis has been considered as possible mechanisms to cause Parkinson’s disease, thereby leading to ERS. MPP^+^ is the active metabolite of MPTP, which functions to stimulate the generation of superoxide radicals *in vitro* and induce cell apoptosis. H_2_S can attenuate the MPTP-induced accumulation of ROS, thereby reducing oxidative stress and ERS. UCP2 can function for H_2_S to reduce ROS production, acting upstream of KATP channels. In addition, H_2_S can directly and indirectly reduce ROS accumulation through the KATP/PI3K/AKT/Bcl-2 pathway.

Lu et al. have demonstrated that H_2_S can attenuate the loss of SN-dense dopamine neurons and the MPTP-induced accumulation of ROS, thereby reducing oxidative stress and ERS. Mitochondrial uncoupling protein 2 (UCP2) can serve as a mechanism for H_2_S to reduce ROS generation. Mitochondrial uncoupling protein 2 (UCP2), which is associated with dopamine neurons, can function for H_2_S to reduce ROS production, acting upstream of KATP channels. In addition, H_2_S can directly and indirectly reduce ROS accumulation through the KATP/PI3K/AKT/Bcl-2 pathway (Lu et al., 2012). Chen’s work shows that appropriate concentrations of H_2_S can protect neurons by maintaining MMP and weakening ROS generation (Chen et al., 2015). MPP^+^ inhibits the production of endogenous H_2_S, so H_2_S not only needs to maintain MMP but also to resist MPP^+^-induced cytotoxicity and apoptosis by reducing ROS accumulation (Tang et al., 2011; Xiao et al., 2016). The pathway that described the above content is shown in **Figure 3**. Therefore, the neuroprotective therapy for PD can exert H_2_S to prevent ERS.

### Neuroprotective Effects of H_2_S in Major Depression Disorder

As people’s psychological pressure gradually increases, the incidence of depression increases year by year. Depression is a common mental disorder. Its clinical features are emotional disorders, discomfort, and despair. Severe cases even have suicide attempts. The etiology of major depressive disorder (MDD) is a combination of multiple factors (Chirita et al., 2015). Neurochemical mechanisms of depression mainly involve the synergistic action of three major neurotransmitter systems: 5-hydroxytryptamine (5-HT), Noradrenaline (NE), and dopamine (DA). According to the World Health Organization estimates, depression places a huge social burden on nonfatal health consequences (Chirita et al., 2015). Therefore, the treatment of MDD is very important. Unfortunately, existing treatment methods cannot prevent the high recurrence of symptoms (Liu et al., 2011). New targets for depression still need to be studied.

The pathogenesis of depression is not yet complete, but some major hypotheses have been proposed: hippocampal neurogenesis and the BDNF/TrkB pathway may be involved in the pathophysiology of depression (Castren and Rantamaki, 2010; Hanson et al., 2011). The neuro-plasticity of MDD is abnormal. BDNF maintains the development of neurons. Under the circumstances of stress, the expression of BDNF may be suppressed, which interrupts the supply of BDNF in the hippocampus. Atrophy or apoptosis of susceptible neurons causes depression and recurrent episodes. Therefore, abnormalities in synaptic plasticity can make MDD worse (Duman, 2002). H_2_S has been reported to enhance neuronal synaptic transmission and promote its long-term enhanced induction (Du et al., 2004). H_2_S can play a positive role in MDD based on its protection of hippocampus and nerve cells. Tan et al. have shown that H_2_S interferes with the process of hippocampal neuron volume reduction and impaired function under stress-induced MDD (Tan et al., 2015). ERS refers to overload caused by ER dysfunction which is a key step in the pathogenesis of neurodegenerative diseases (Stefani et al., 2012). ERS links to the pathogenesis of depression caused by chronic unpredictable mild stress (CUMS). Amphetamine and inhibition of rat brain striatum stress can activate transcription of ERS transcription factors ATF3 and ATF4 (Pavlovsky et al., 2013). H_2_S not only attenuates homocysteine-induced apoptosis in hippocampal neurons and ERS by upregulating the expression of BDNF-TrkB in the MDD model but also improves CUMS-induced depression and inhibits hippocampus by promoting the expression of hippocampal Sirt-1 ERS (Wei et al., 2014; Liu et al., 2017).

**Figure 4:**
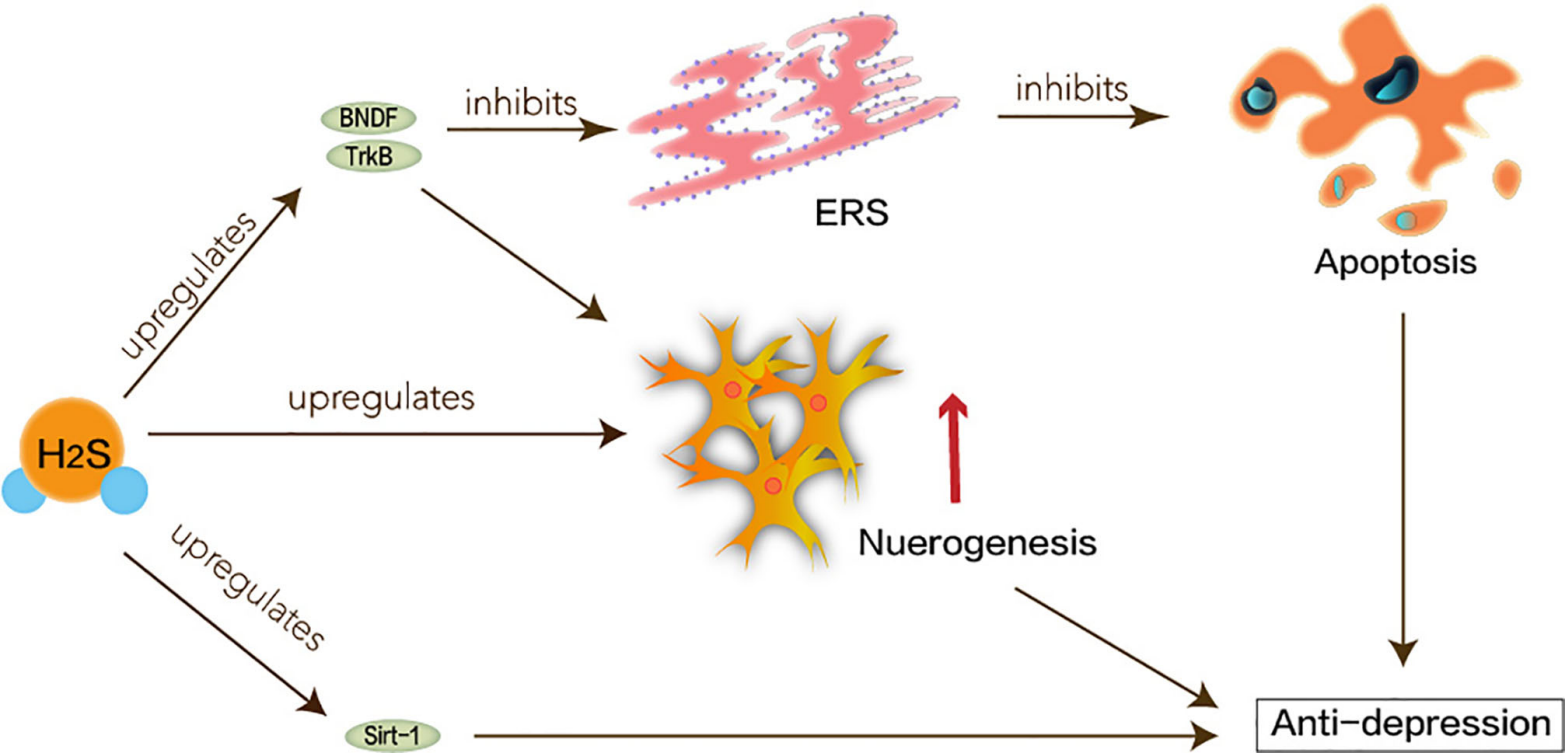
Neuroprotective effects of H_2_S in major depression disorder. ERS linked to the pathogenesis of depression caused by CUMS. Hippocampal neurogenesis and the BDNF/TrkB pathway may be involved in the pathophysiology of depression. H_2_S interferes with the process of hippocampal neuron volume reduction and impaired function. H_2_S not only attenuates homocysteine-induced apoptosis in hippocampal neurons and ERS by upregulating the expression of BDNF-TrkB in the MDD model, but also improves CUMS-induced depression and inhibits hippocampus by promoting the expression of hippocampal Sirt-1 ERS.

Taken together, as shown in **Figure 4**, H_2_S signaling molecules in the brain can understand new antidepressant pathways and mechanisms through ERS.

## Conclusion and Perspectives

Different studies have recognized that H_2_S plays an important role in physiological and pathological conditions in the body. Appropriate concentration of H_2_S has a protective and regulatory role in the CNS, existing in cells in free, acid-labile, and bound sulfane sulfur form. Free exogenous H_2_S is able to exert a physiologic function in neurotransmission and cell survival. Although the neuroprotective effects of H_2_S are mainly emphasized in this article, either excessive or insufficient H_2_S still has pathogenic effects on various systems. Excessive H_2_S initiates neuro-cytotoxic mechanism in the brain. Cells and tissues need to maintain appropriate concentrations of H_2_S to prevent potential toxicity. There is no doubt that the misfolding of proteins and the accumulation of unfolded proteins in the ER cavity trigger neurotoxic effects. The overload of ER activates the ERS pathway and plays a role in the pathogenesis of a series of neurological diseases. Potential mechanisms that trigger the ERS response may be closely related to toxic levels of homocysteine, oxidative stress, and abnormal epigenetic modification. H_2_S can regulate the expression of various proteins and genes under the condition of ERS,and maintain the homeostasis of cells *in vivo*. It is considered using H_2_S to directly or indirectly target drug-mediated treatment of CNS diseases by modulating the ERS mechanism. One challenge of H_2_S-based therapeutics is its delivery development. H_2_S has attractive applications in neurological diseases and psychiatry. H_2_S will be a promising agent for neurodegenerative diseases.

## Author Contributions

HZ and HY discussed the concepts and wrote the manuscript. JC, JS, LG, PH, and YZ reviewed the literature and provided critical revision of the manuscript for important content.

## Funding

This study received support from the Shanghai Municipal Health and Family Planning Commission Traditional Chinese Medicine Research Project Foundation (No.2018JP008).

## Conflict of Interest

The authors declare that the research was conducted in the absence of any commercial or financial relationships that could be construed as a potential conflict of interest.
